# Physiological cerebrospinal fluid interactions between brain and eye structures are altered after long‐duration spaceflight

**DOI:** 10.1113/EP093112

**Published:** 2026-01-16

**Authors:** Ge Tang, Steven Jillings, Ben Jeurissen, Elena Tomilovskaya, Inna Nosikova, Alexandra Ryabova, Ekaterina Pechenkova, Viktor Petrovichev, Ilya Rukavishnikov, Stefan Sunaert, Paul M. Parizel, Lyudmila Makovskaya, Valentin Sinitsyn, Jan Sijbers, Jitka Annen, Steven Laureys, Angelique Van Ombergen, Seyed‐Ahmad Ahmadi, Floris L. Wuyts, Peter zu Eulenburg

**Affiliations:** ^1^ Institute for Neuroradiology University Hospital LMU Munich Munich Germany; ^2^ Graduate School of Systemic Neurosciences LMU Munich Munich Germany; ^3^ Lab for Equilibrium Investigations and Aerospace University of Antwerp Antwerp Belgium; ^4^ Imec/Vision Lab University of Antwerp Antwerp Belgium; ^5^ SSC RF – Institute of Biomedical Problems Russian Academy of Sciences Moscow Russia; ^6^ Laboratory for Cognitive Research HSE University Moscow Russia; ^7^ Radiology Department Federal Centre of Treatment and Rehabilitation Moscow Russia; ^8^ Translational MRI Department of Imaging & Pathology KU Leuven Leuven Belgium; ^9^ Radiology Department Antwerp University Hospital & University of Antwerp Antwerp Belgium; ^10^ Faculty of Fundamental Medicine Lomonosov Moscow State University Moscow Russia; ^11^ Faculty of Psychology and Educational Sciences Department of Data Analysis Ghent University Ghent Belgium; ^12^ Coma Science Group, GIGA‐Consciousness University of Liège Liège Belgium; ^13^ Centre du Cerveau^2^ University Hospital of Liège Liège Belgium; ^14^ Department of Translational Neurosciences – ENT University of Antwerp Antwerp Belgium

**Keywords:** cerebrospinal fluid, long‐duration spaceflight, magnetic resonance imaging, periorbital morphology

## Abstract

Long‐duration spaceflight represents an extreme challenge, triggering adaptive responses including spaceflight‐associated neuro‐ocular syndrome, characterized by diminished visual acuity and ocular changes, which is a significant health risk for Mars missions. Concurrently, spacefarers experience brain adaptations, including ventricular expansion and CSF redistribution; however, the integrative physiological mechanisms linking these brain–eye responses remain unestablished. We applied automated morphometric analysis to investigate brain–eye relationships using high‐resolution MRI data from terrestrial control subjects and spacefarers, conducting correlation analysis between third ventricle volume and ocular parameters. Analysis revealed significant baseline correlations between third ventricle volume and multiple ocular parameters in healthy control subjects, including globe dimensions, optic nerve sheath volume, optic nerve length and retro‐orbital width. Following spaceflight, adaptations occurred: optic nerve sheath volume increased by 11.93 ± 6.07 mm^3^ (right) and 27.22 ± 8.74 mm^3^ (left), and optic nerves lengthened by 0.38 mm (right) and 0.47 mm (left). The baseline analysis showed that multiple orbital structures were correlated with third ventricle volume, whereas longitudinal change analysis revealed selective associations: only globe changes were correlated with third ventricle expansion. This study demonstrates structure‐specific brain–eye relationships consistent with CSF‐mediated coupling as a mechanism underlying spaceflight‐associated neuro‐ocular syndrome during the cranial microgravity response. Following spaceflight, third ventricle expansion is correlated selectively with globe changes, while dissociating from optic nerve sheath and retro‐orbital adaptations, providing quantitative evidence that microgravity creates differential biomechanical effects across orbital compartments. This supports CSF compartmentalization and the limited intracranial volume expansion (cranial ceiling effect) as underlying mechanisms. The findings illuminate the limits of brain elastic tissue expansion during intracranial fluid accumulation in astronauts.

## Abstract (highlights)

- **What is the central question of this study?**
  Do microgravity‐induced changes in and behind the eye represent local adaptations or reflect a head‐wide phenomenon coupling brain–eye physiology through CSF redistribution?
- **What is the main finding and its importance?**
  Brain–eye correlations were established at baseline. Following long‐duration spaceflight, third ventricle expansion exhibits selective coupling with globe morphometric changes while dissociating from retro‐orbital structural adaptations. This supports CSF compartmentalization combined with limited intracranial volume expansion (cranial ceiling effect) as mechanisms. Eye structural changes are part of the cranial microgravity response. The findings inform countermeasures targeting cephalad fluid shift for Mars missions.

## 1 INTRODUCTION

Living in space under microgravity has profound and wide‐ranging impacts on human physiology, with spaceflight‐associated neuro‐ocular syndrome (SANS) emerging as one of the most significant health risks for future exploratory missions. During long‐duration spaceflight aboard the International Space Station (ISS), astronauts experience diminished near‐distance visual acuity, which begins ∼6 weeks into the mission and persists after return to Earth. Clinical examinations reveal persistent orbital and retro‐orbital changes, including optic disc oedema, globe flattening, choroidal folds and nerve fibre thickening, often manifesting asymmetrically, with right‐eye predominance (Mader et al., 2011). Cumulative quantitative evidence over the past decade has documented structural alterations across multiple orbital compartments, including changes in the cross‐sectional area of the optic nerve sheath (ONS), lengthening of the optic nerve (ON), altered tortuosity, volumetric displacement of the globe and modifications to retro‐orbital tissue that collectively define the morphological signature of SANS (Rohr et al., 2020; Sater et al., 2021; Scott et al., 2020; Wahlin et al., 2021).

Originally termed visual impairment intracranial pressure syndrome, these changes were attributed to elevated intracranial pressure (ICP) (Van Ombergen et al., 2019) based on similarities to terrestrial pathological status, such as idiopathic intracranial hypertension, in which patients exhibit symmetrically extended ONS, an altered ON vertical tortuosity, optic nerve head protrusion and a concave deformation of the pituitary gland as common structural changes (Bidot et al., 2015; D'Antona et al., 2021; Ng & Mollan, 2025). However, most of the space crew seem to develop vision disorders asymmetrically in the beginning (Mader et al., 2017), contrasting with the bilateral manifestations typical of elevated ICP disorders, which challenged this interpretation and led to the current SANS terminology, emphasizing the unclear pathophysiology and CNS involvement. This complexity has generated two competing mechanistic hypotheses: elevated ICP causing uniform pressure increases throughout the cranial–orbital system versus CSF compartmentalization creating localized pressure effects within specific orbital regions (Rodrigues et al., 2025). The latter also gives rise to other related localized mechanisms that have been proposed, such as regional glymphatic stasis and oxidative stress within orbital compartments (Ong et al., 2022; Wojcik et al., 2020).

Concurrent with ocular changes, long‐duration spaceflight produces significant brain structural adaptations, including CSF redistribution and ventricular expansion. Roberts et al. (2017) first depicted the narrowing of the CSF space at the vertex in the US cohort of astronauts. One year later, Van Ombergen and colleagues also discovered increased CSF in the ventral space and a decrease in the vertex area post‐flight, indicating the upward shift of the brain. Notably, the third ventricle showed a 12.9% increase in volume post‐flight in the Russian cosmonauts compared with pre‐flight measurements (Van Ombergen et al., 2018, 2019). However, the relationship between ICP and ventricular volume in the microgravity environment remains unknown, because direct ICP measurements in spacefarers aboard the ISS are currently impossible. Despite extensive documentation of both brain and ocular changes through previous studies using visual inspection and semi‐automatic quantitative measurements for the ocular region (Mader et al., 2011; Rohr et al., 2020; Sater et al., 2021; Wostyn & De Deyn, 2018), the physiological relationship linking these adaptations has remained unestablished. We think that eye and brain changes that occur in long‐duration missions (>40 days) in microgravity are essentially linked by a general cranial microgravity response (Seidler et al., 2024; Zu Eulenburg et al., 2025). Rather than assuming a specific direction of correlation, we analysed whether ventricular volume, especially third ventricle volume, and ocular and retro‐orbital morphology demonstrate systematic relationships that would indicate coordinated intracranial changes versus isolated effects within the retro‐orbital region, providing evidence to investigate ocular and brain changes comprehensively.

To address this fundamental gap, we investigated the intricate associations between ocular and retro‐orbital structures and intracranial ventricular compartments using automated morphometric analysis, with the specific goal of a quantitative assessment of brain–eye coupling patterns.

Our approach examines both baseline brain–eye relationships in healthy populations and their selective dissociation following spaceflight exposure, providing insights into potentially CSF‐mediated mechanisms underlying coordinated structural adaptations across the brain–eye system and for developing targeted countermeasures for future Mars missions.

## 2 MATERIALS AND METHODS

### 2.1 Cohort demographics

This prospective cross‐sectional study includes brain MRI data collected according to a scientific protocol in a European Space Agency (ESA)‐endorsed, prospective MRI study (‘BRAIN‐DTI’) involving Russian Orbital Service Station (ROS) cosmonauts and ESA astronauts, in addition to concomitant control participants for each group of spacefarers and study site. Cranial MRI scans were acquired in 13 male ROS cosmonauts (and a small sample of ESA astronauts; for details on this cohort, please see the Supporting Information) who participated in long‐duration missions to the ISS. The ROS cosmonauts were scanned with a mean (SD) of 88.8 (32.6) days before launch to the ISS (pre‐flight MRI) and 9.5 (3.0) days after return to Earth (post‐flight MRI). The mean (SD) mission duration was 184.8 (52.6) days. Brain MRI data were also acquired in 15 healthy ROS control participants, who were of a similar age and education level to the space crews and who were scanned at a comparable interval to account for age‐related effects. The average demographic and time interval characteristics of the participants are reported in Table 1.

**TABLE 1:** Demographics of both long‐duration spaceflight cohorts.

Parameter	Cosmonauts (13)	Control subjects (cosmonauts) (15)	Astronauts^a^	Control subjects (astronauts) (8)
Age at pre‐flight MRI scan, years	45.5 ± 5.3	42.3 ± 6.3	^a^	39.0 ± 3.5
Pre‐flight MRI scan before launch, days	88.8 ± 32.6	–	137.2 ± 77.2	–
Mission duration, days	184.8 ± 52.6	–	Similar to cosmonauts^a^	–
Post‐flight MRI scan after return, days	9.5 ± 3.0	–	4.8 ± 2.9	–
Pre‐ and post‐flight interval, days	283.1 ± 57.0	226.6 ± 66.8	333.4 ± 70.1	164.6 ± 39.1
Follow‐up MRI scan after return, days	233.6 ± 55.8	–	194.3 ± 25.9	–
Pre‐flight and follow‐up interval, days	511.4 ± 95.4	495.8 ± 138.0	499.8 ± 59.5	362.5 ± 45.9
Previous days in space, days	196.8 ± 191.0	–	^a^	–

^a^ Some demographics and mission information, in addition to the final sample size, were withheld at the request of the European Space Agency to protect astronaut corps anonymity.

In this paper, we report the results from the cohort of cosmonauts. Owing to the small sample size for data from ESA astronauts, we included their supportive findings as complementary data in the Supporting Information as preliminary results (see Supporting Information Table S2).

### 2.2 Ethical approval

The study was approved by the ESA medical board, by the Institutional Review Board of the Antwerp University Hospital (13/38/357), by the Committee of Biomedicine Ethics of the Institute of Biomedical Problems of the Russian Academy of Sciences and by the Human Research Multilateral Review Board (HRMRB). All participants, cosmonauts, astronauts and healthy control subjects, provided written informed consent, and the investigations adhered to the principles outlined in the *Declaration of Helsinki* and its subsequent amendments.

### 2.3 MRI protocol

All head scans were performed on clinical 3 T MRI machines equipped with a multi‐channel receiver head coil. The scientific MRI protocol included the acquisition of three‐dimensional high‐resolution T_1_‐weighted structural images (voxel size 1 mm × 1 mm × 1 mm). ROS cosmonauts and control subjects were scanned on a GE Discovery MR750 3T MRI system (GE Healthcare, Milwaukee, WI, USA) at the National Medical Research Treatment and Rehabilitation Centre of the Ministry of Health of Russia in Moscow, Russia, with the following sequence parameters: T_1_‐weighted fast spoilt gradient echo; 176 slices; Repetition Time = 7.9 ms; Echo Time = 3.06 ms; Inversion Time = 450 ms; and flip angle = 12°. The ESA astronauts and their respective control subjects were assessed on a dedicated combined 3 T MRI/PET research scanner (Siemens Biograph, Erlangen, Germany) located at the ENVIHAB facility in Cologne, Germany, using a 20‐channel head and neck array coil. For each time point, two high‐resolution sagittal T_1_‐weighted three‐dimensional magnetization prepared rapid gradient echo (MPRAGE) images were acquired ∼1 h apart (176 slices; Repetition Time = 1900 ms; Echo Time = 2.43 ms; Inversion Time = 900 ms; voxel size 1 mm × 1 mm × 1 mm; flip angle 9°; field of view = 256 mm; bandwidth 180 Hz per pixel).

### 2.4 MRI data quality assurance and brain segmentation

Data quality assurance (sample homogeneity), data preprocessing and analysis were performed with the CAT12 toolbox (v.2170) by Christian Gaser and Robert Dahnke (Department of Psychiatry, University of Jena, Jena, Germany; http://www.neuro.uni‐jena.de/cat) within the framework of Statistical Parametric Mapping SPM12 (v.7771) (http://www.fil.ion.ucl.ac.uk/spm; Wellcome Department of Cognitive Neurology, London, UK), using Matlab R2021b (Mathworks, Natick, MA, USA) (Gaser et al., 2024).

The data were processed using the longitudinal preprocessing option of CAT12 for large effects without priors, where spatial normalization parameters are calculated using an average image of the two time points. This was again applied to the first, second and third images, respectively. Afterwards, we derived tissue volume estimation in native space using the ventricle regions of interest (ROIs) from the Neuromorphometric atlas (provided by Neuromorphometrics, Inc.; http://neuromorphometrics.com). Total intracranial volume as a surrogate for head size, in addition to total grey matter, white matter and CSF volume, was computed. Four ROIs applicable to the CSF space were included: the lateral ventricles (left and right), the third ventricle and the fourth ventricle. These ROIs, which are defined in Montreal Neurological Institute template space, were transformed to native subject space for the volume calculations using the inverse non‐linear deformations needed to spatially normalize images to Diffeomorphic Anatomical Registration using Exponentiated Lie algebra template space with the geodesic shooting algorithm.

For a separate deformation‐based analysis (deformation‐based morphometry), which looks at local volume changes independent of tissue type, the T_1_‐weighted images were segmented with an adaptive maximum a posteriori (AMAP) algorithm after spatial adaptive non‐local means denoising and normalized into Montreal Neurological Institute space at 1 mm resolution by means of strong geodesic shooting using an existing IXI template (https://brain‐development.org/ixi‐dataset/). The normalized Jacobian determinants for deformation‐based morphometry were generated. These normalized images of the determinants were then smoothed with an isotropic 6 mm full width at half maximum Gaussian kernel. The subsequent statistical analyses were conducted using SPM12. A within‐subject ANOVA approach was used to test for differences between the time points. The respective T‐contrasts were considered significant using threshold‐free cluster enhancement, with 10 000 permutations and a cluster threshold of *P* < 0.05, including a family‐wise error rate multiple testing correction. The deformation‐based morphometry results were visualized with MRIcroGL (version 1.0, 2019; Chris Rhorden) (https://www.nitrc.org/projects/mricrogl/) as overlays onto the IXI‐555 template.

### 2.5 Ocular morphometry

**FIGURE 1:**
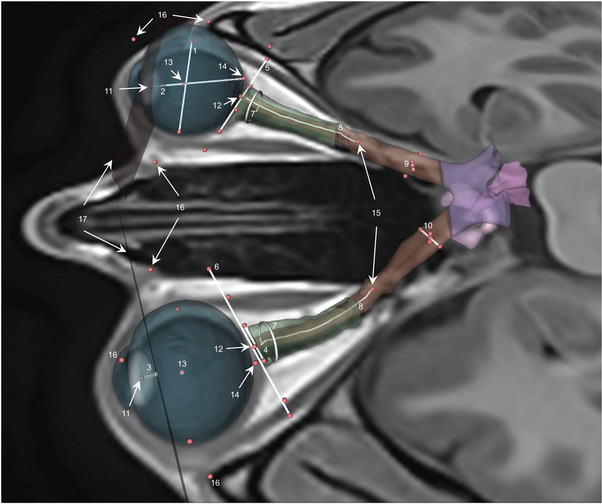
Segmentation planes and landmarks in the template. Overview of all the measured parameters for the eye, retro‐orbital space and optic nerve with respect to the landmarks (red dots), as follows: 1, globe width; 2, globe length; 3, perpendicular point‐to‐plane distance of lens centre to the orbital rim (plane estimated with least‐squares fit); 4, ONS head width; 5, retro‐orbital width (muscle); 6, retro‐orbital width (bone); 7, 3 mm intersection area (ONS 3 mm); 8, ON centre line; 9, optic canal minor axis; 10, optic canal major axis; 11, centre of the lens; 12, ON tip; 13, centre of the globe; 14, back of the globe; 15, end of the optic canal; 16, points on bony orbital rim; 17, estimated orbital rim plane (dark pink) (adapted from MReye‐Seg, https://github.com/Eulenburg‐LMU/MReye‐Seg). Abbreviations: ON, optic nerve; ONS, optic nerve sheath.

Ocular morphometric parameters were extracted using the MReye‐Seg pipeline, previously validated for standardized orbital assessment (https://github.com/Eulenburg‐LMU/MReye‐Seg) (Tang et al., 2025) (The intraclass correlation coefficients for validation of this dataset are reported in Support Information Table S1). This automated workflow integrates orbital template generation from structural MRI, expert‐guided segmentation of ocular and retro‐orbital structures with anatomical landmark annotation, registration‐based propagation to individual scans, quality assurance protocols, and automated extraction of bilateral morphometric parameters. The pipeline has demonstrated high reproducibility in previous validation studies across multiple datasets. The specific ocular and retro‐orbital measurements analysed in this study are illustrated in Figure 1.

### 2.6 Statistical analysis

Our analytical approach prioritized detection of pre‐ and post‐flight brain structure changes within each group separately, rather than using mixed ANOVA, owing to the limited sample size of the spacefarer cohort (*n* = 13). This decision maximized statistical power for identifying significant structural alterations while enabling our secondary research goal, i.e., examining the correlation between ventricular and ocular changes following spaceflight. The control group served as a reference for normal temporal variation rather than for direct statistical comparison.

For the primary analysis, we applied Student's paired *t*‐tests to assess pre‐ to post‐flight differences in all morphometric parameters. For correlation analysis between general brain volume, ventricular volume and ocular metrics, we calculated the Pearson correlation coefficient. Given the exploratory nature of this discovery study with a limited sample size, we did not apply a false discovery rate correction; instead, we report unadjusted *P*‐values to maximize the sensitivity for detecting potential relationships that warrant further investigation in larger validation cohorts (Nicholson et al., 2022; Rubin, 2017).

## 3 RESULTS

**FIGURE 2:**
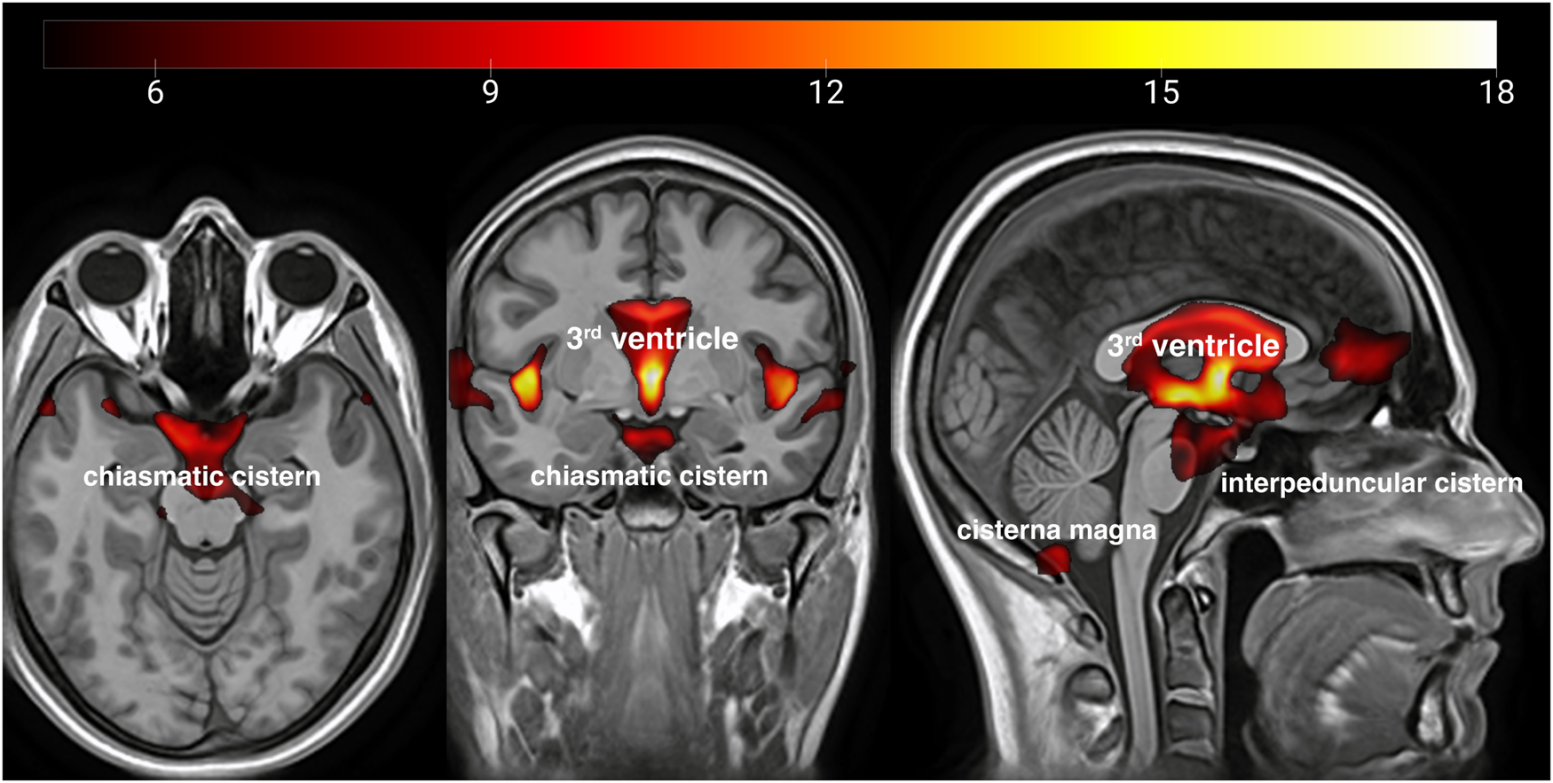
Deformation‐based morphometry findings in long‐duration spaceflight cosmonauts. The depicted post‐flight differences compared with baseline highlight the substantial volumetric changes occurring after exposure to 6 months of microgravity in this tissue‐type agnostic approach. The peak increases are predominantly found in the cisterns and ventricles of the CSF compartments situated around the geometric centre of the head. The most significant change of the Jacobian determinant, indicating any tissue expansion, is in and around the third ventricle, whilst also strongly affecting the nearby suprasellar and chiasmatic cisterns. The affected cisterns are directly proximal to the funnel entry point of both optic canals. The scale reflects *t*‐values (*P *< 0.01 family‐wise error).

We conducted several analyses on ocular and retro‐orbital parameters in conjunction with separately segmented CSF volume information (Figure 2). All results, except for the final descriptive statistics regarding post‐flight CSF volume dynamics, were derived from the original cohort of cosmonauts and their respective control subjects.

### 3.1 General correlation of ocular and retro‐orbital morphometrics data

**FIGURE 3:**
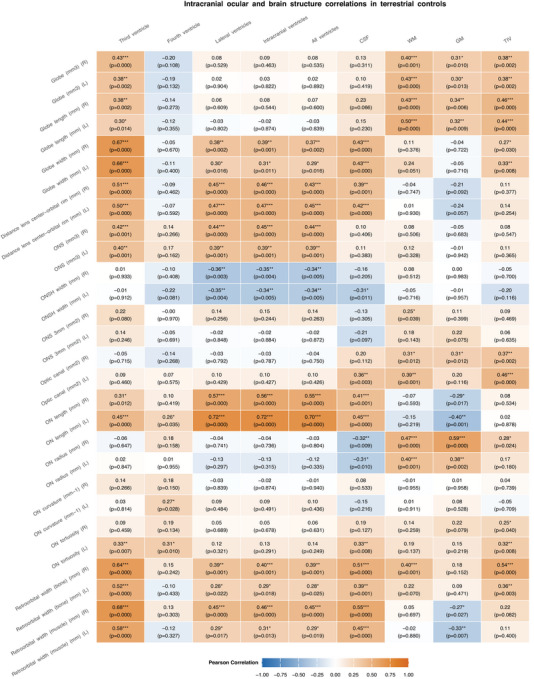
Heatmap depicting the physiological correlations between ocular and brain morphometrics in the terrestrial control group. Pearson correlation coefficients between ocular, ventricular and general brain volumetrics were derived from the Earth‐based control subjects (*n* = 66). Each cell contains the correlation coefficient (*r*) and the corresponding *P*‐value. Statistical significance is indicated as follows: ^*^ *P* < 0.05, ^**^ *P* < 0.01 and ^***^ *P* < 0.001. Blue indicates a negative and red a positive correlation.

Based on our previous findings, which demonstrate substantial volumetric changes in the third ventricle following spaceflight (Van Ombergen et al., 2019), we selected this structure as our primary ventricular reference parameter for correlation analyses with ocular structures in the control group. The deformation maps in Figure 2 illustrate the expansion of the third ventricle and its effects on adjacent suprasellar and chiasmatic cisterns, confirming its central role in spaceflight‐induced CSF redistribution. Correlation analysis revealed significant associations between third ventricle volume and multiple globe properties {right globe volume: *r*(64) = 0.43, *P* = 0.0003, 95% confidence interval (CI) [0.21, 0.61]; left globe volume: *r*(64) = 0.38, *P* = 0.0017, 95% CI [0.15, 0.57]; right globe length: *r*(64) = 0.38, *P* = 0.0015, 95% CI [0.16, 0.57]; left globe length: *r*(64) = 0.3, *P* = 0.0143, 95% CI [0.06, 0.51]; right globe width: *r*(64) = 0.67, *P* < 0.0001, 95% CI [0.51, 0.79]; left globe width: *r*(64) = 0.66, *P* < 0.0001, 95% CI [0.5, 0.78]} and the retro‐orbital structures {right ONS volume: *r*(64) = 0.42, *P* = 0.0005, 95% CI [0.19, 0.6]; left ONS volume: *r*(64) = 0.40, *P* = 0.001, 95% CI [0.17, 0.58]; right ON length: *r*(64) = 0.31, *P* = 0.012, 95% CI [0.07, 0.51]; left ON length: *r*(64) = 0.45, *P* = 0.0001, 95% CI [0.24, 0.63]; right retro‐orbital width (bone): *r*(64) = 0.64, *P* < 0.0001, 95% CI [0.47, 0.76]; left retro‐orbital width (bone): *r*(64) = 0.52, *P* < 0.0001, 95% CI [0.31, 0.67]; right retro‐orbital width (muscle): *r*(64) = 0.68, *P* < 0.0001, 95% CI [0.52, 0.79]; left retro‐orbital width (muscle): *r*(64) = 0.58, *P* < 0.0001, 95% CI [0.39, 0.72]}. In addition, we observed robust correlations between total brain volume and retro‐orbital anatomical features, particularly within the retro‐orbital width and optical canal {right optical canal: *r*(64) = 0.37, *P* = 0.002, 95% CI [0.14, 0.56]; left optical canal: *r* (64) = 0.46, *P* < 0.0001, 95% CI [0.24, 0.63]}. For a detailed overview of the correlations, please see Figure 3.

### 3.2 Differences in ocular and retro‐orbital structure post‐flight

**FIGURE 4:**
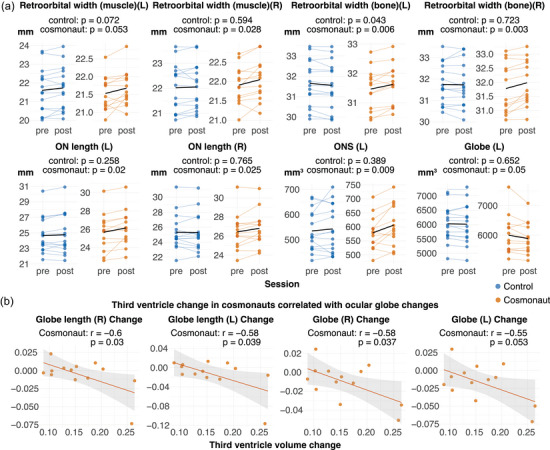
Longitudinal ocular changes following long‐duration spaceflight and correlations with third ventricle volume expansion. (a) Pre‐ to post‐flight changes in ocular metrics among long‐duration spaceflight cosmonauts, displayed for each eye. Blue lines indicate individual pre‐ and post‐flight measurements, while orange lines represent the collective cosmonaut cohort trend. Black lines represent mean trajectories. (b) Relationship between third ventricle volume changes and ocular metric changes in cosmonauts. The scatterplots display individual measurements with regression lines (±95% confidence intervals) for cosmonauts. Correlation coefficients (*r*) and corresponding *P*‐values are shown for each group. Abbreviations: L, left; ON, optic nerve; ONS, optic nerve sheath; R, right.

For the cosmonaut group, Student's paired *t*‐test showed significant differences between the pre‐ and post‐flight measurements in multiple parameters for both eyes alike (see Figure 4a). At the same time, we found a significant increase in the retro‐orbital width (bone) post‐flight compared with the pre‐flight baseline {right eye: mean difference ± SEM = 0.2 ± 0.06 mm, *t*(12) = 3.65, *P* = 0.003, *d* = 1.01, 95% CI [0.08, 0.33]; left eye: mean difference ± SEM = 0.16 ± 0.05 mm, *t*(12) = 3.35, *P* = 0.006, *d* = 0.93, 95% CI [0.05, 0.26]}, accompanied by an increase of the retro‐orbital width (muscle) post‐flight, especially in the right eye {right eye: mean difference ± SEM = 0.14 ± 0.06 mm, *t*(12) = 2.50, *P* = 0.028, *d* = 0.69, 95% CI [0.02, 0.26]; left eye: mean difference ± SEM = 0.15 ± 0.07 mm, *t*(12) = 2.15, *P* = 0.053, *d* = 0.6, 95% CI [0.0, 0.3]}. The same directional change, a volume increase, was detected in both ONS, with a clear increase on the left side and a marginal increase on the right side {right ONS: mean difference ± SEM = 11.93 ± 6.07 mm^3^, *t*(12) = 1.96, *P* = 0.073, *d* = 0.54, 95% CI [−1.31, 25.16]; left ONS: mean difference ± SEM = 27.22 ± 8.74 mm^3^, *t*(12) = 3.11, *P* = 0.009, *d* = 0.86, 95% CI [8.17, 46.27]}. The length of the ON also increased significantly post‐flight {right ON: mean difference ± SEM = 0.38 ± 0.15 mm, *t*(12) = 2.55, *P* = 0.025, *d* = 0.71, 95% CI [0.06, 0.7]; left ON: mean difference ± SEM = 0.47 ± 0.17 mm, *t*(12) = 2.68, *P* = 0.020, *d* = 0.74, 95% CI [0.09, 0.85]}. The total globe volume decreased slightly post‐flight in both eyes, although we can see a larger decrease in the left globe volume {right eye: mean difference ± SEM = −71.29 ± 38.98 mm^3^, *t*(12) = −1.83, *P* = 0.092, *d* = 0.51, 95% CI [−156.21, 13.63]; left eye: mean difference ± SEM = −112.16 ± 51.50 mm^3^, *t*(12) = −2.18, *P* = 0.050, *d* = 0.6, 95% CI [−224.36, 0.05]}. After removal of one outlier, who showed a significant backward globe movement (right eye: 1.26 mm; left eye: 1.67 mm), all the 12 other spacefarers showed a significant forward movement in the left globe, while the right globe showed a non‐significant forward displacement {right eye: mean difference ± SEM = 0.22 ± 0.12 mm, *t*(11) = 1.81, *P* = 0.098, *d* = 0.52, 95% CI [−0.05, 0.49]; left eye: mean difference ± SEM = 0.43 ± 0.15 mm, *t*(11) = 2.87, *P* = 0.015, *d* = 0.83, 95% CI [0.1, 0.77]}.

### 3.3 The magnitude of observable volumetric change in the third ventricle is associated with a concurrent change in ocular morphometrics

To further explore these relationships, we investigated the association between pre‐ to post‐flight changes in third ventricle volume and concurrent changes in ocular morphometrics in the group of cosmonauts. Here, we observed that the expansion of the third ventricle was correlated with a larger decrease of the globe length in each eye post‐flight {right eye: *r*(11) = −0.60, *P* = 0.030, 95% CI [−0.86, −0.07]; left eye: *r*(11) = −0.58, *P* = 0.039, 95% CI [−0.86, −0.04]}. A greater expansion of the third ventricle was also associated with a larger decrease in total right globe volume {right eye: *r*(11) = −0.58, *P* = 0.037, 95% CI [−0.86, −0.05]; left eye: *r*(11) = −0.55, *P* = 0.053, 95% CI [−0.84, 0.01]}.

Finally, the increase in third ventricle expansion trended to be correlated with a marginally greater post‐flight increase of the left but not the right eye in ONS head width {right eye: *r*(11) = 0.15, *P* = 0.629, 95% CI [−0.44, 0.65]; left eye: *r*(11) = 0.52, *P* = 0.071, 95% CI [−0.05, 0.83]} and retro‐orbital width (muscle) {right eye: *r*(11) = −0.05, *P* = 0.869, 95% CI [−0.59, 0.51]; left eye: *r*(11) = 0.54, *P* = 0.057, 95% CI [−0.02, 0.84]}. For a detailed depiction and description, see Figure 4b.

### 3.4 Deformation map changes of the posterior globe

**FIGURE 5:**
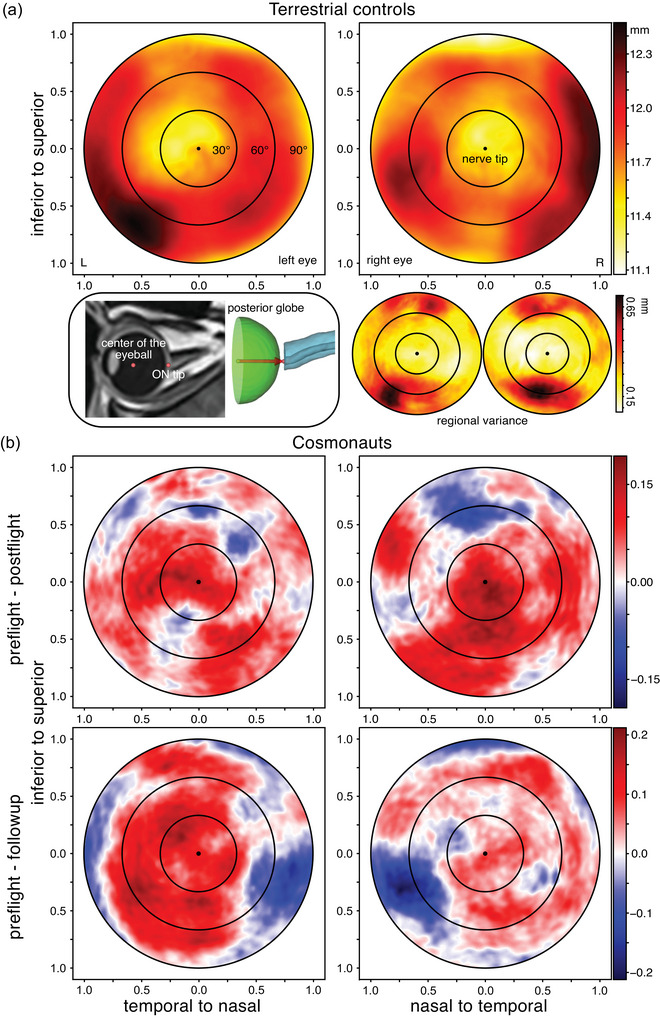
Observed deformation maps of the posterior globe in long‐duration spaceflight. These contour maps (0°–90°) represent the radius measurements in a spherical coordinate system for the entire posterior half of the globe. The radius is calculated from the geometric centre of the globe to the scleral wall, depicted with the centre of the optic nerve (ON) tip as a reference point and defining vector for a plane perpendicular to this vector. (a) The mean radius in the control subjects was greatest for the very temporal regions (60°–90°) across the upper and lower quadrants of the maps, and in the mid‐to‐lower quadrant of the nasal regions for each eye. The main sources of variance for the radius in the lower > upper third of the deformation map might reflect the insertion zones of the superior rectal and inferior oblique eye muscles in this projection. (b) A shortening of the radius post‐flight (red) can be found in cosmonauts for the area around the ON and lateral to it (right > left eye). This change in shape of the globe persists in the long‐term follow‐up data, but seemingly now more for the left than the right eye. All scales reflect millimetres.

A deformation map is the visual representation of the changes in the shape of the posterior globe. From our control cohort, we could allow for precise quantification of the deviation of the globe from a perfectly spherical shape. The central region, expanding less than the 30° polar angle, exhibited a relatively shorter radius. For the left eye, the longest radius was in the temporal inferior region, whereas for the right eye it was found in the temporal area. The variance map showed the largest variation for the left eye in the same area with the longest radius, i.e., the temporal inferior area. Conversely, the maximum variance for the right eye was observed in the nasal inferior area (see Figure 5).

The deformation map post‐flight showed globe flattening, especially in the central region, which spans less than a 30° polar angle. The right globe presented a more pronounced degree of globe flattening than the left one. The deformation map of the follow‐up also showed globe flattening. Interestingly, the left posterior globe showed a higher degree of deformation, predominantly in the temporal area.

### 3.5 The magnitude of observable structural changes for the brain and eye with respect to the time point of data collection post‐flight (pooled analysis from the combined cohort of cosmonauts and astronauts)

We also investigated a potential role for the delay in post‐flight scanning in previously published pre‐ versus post‐flight differences for the ventricular CSF system, and for the ONS volume and the optical canal size. In general, from our dataset, we can plot a linear function, which highlights that the later the scan post‐flight (range 1–14 days after return to Earth), the smaller the pre‐ versus post‐flight differences for the ONS, and the larger the differences for the lateral ventricle and the fourth ventricle. However, this finding is not significant and warrants further investigation with larger datasets in the future. For the third ventricle, we also could not observe a significant confounder effect of scanning day post‐flight within the first 14 days (see Supporting Information Figure S1).

## 4 DISCUSSION

This study addressed a critical knowledge gap in spaceflight‐associated brain and eye research by investigating the relationship between ventricular changes and ocular morphometric alterations in terrestrial control subjects and spacefarers following long‐duration spaceflight. We established significant baseline correlations between third ventricle volume and multiple ocular parameters in healthy control subjects, including globe dimensions, ONS volume, ON length and retro‐orbital width. Following spaceflight, we documented substantial ocular and retro‐orbital structural changes, including a decreased left globe volume with forward displacement, increased ONS volume, lengthened ON and an expanded retro‐orbital width. Crucially, although spaceflight‐induced morphological changes were observed across multiple orbital structures, the magnitude of third ventricle volume expansion demonstrated selective correlations exclusively with globe morphometric alterations, specifically globe length and volume. Deformation mapping revealed characteristic deformation patterns in the posterior globe segment, showing greater deformation in the 30° posterior region post‐flight. Finally, post‐flight tracking within 14 days post‐flight demonstrated only a descriptive trend towards a decrease in bilateral ONS volume over time, while the increase in third ventricle volume remained, providing an insight into the dynamic relationship between CSF dynamics and ocular structural adaptations during post‐spaceflight recovery.

### 4.1 Ocular morphometric alterations after long‐duration spaceflight

Traditional quantification of spaceflight‐induced ocular changes has relied historically on manual measurement of optic nerve sheath diameter at a standardized 3 mm retrobulbar location (Bäuerle et al., 2013; Helmke & Hansen, 1996). This approach introduces significant observer variability and demonstrates fundamental limitations in capturing the complex three‐dimensional nature of CSF‐related morphological adaptations. This diameter‐based measurement approach assumes uniform ONS expansion and fails to account for the heterogeneous spatial distribution of CSF pressure effects along the ONS. Our implementation of an observer‐independent automated processing pipeline enabled comprehensive volumetric analysis of ocular and retro‐orbital structures, allowing for direct comparison with existing literature through replication of traditional measurements. We observed no significant differences in ONS 3 mm cross‐sectional measurements in our cohort (right ONS 3 mm: mean difference ± SEM = −0.43 ± 0.89 mm^2^, *P* = 0.640; left ONS 3 mm: mean difference ± SEM = −0.01 ± 2.04 mm^2^, *P* = 0.995). These null findings align with the mixed results documented in recent SANS literature and are likely to reflect the inherent limitations of diameter and cross‐sectional area measurements on native T_1_‐weighted images, which lack sufficient sensitivity for detecting the subtle but clinically significant volumetric changes within the ONS compartment (Fall et al., 2022; Kramer et al., 2012; Rohr et al., 2020; Sirek et al., 2014).

However, our approach revealed more comprehensive spaceflight‐induced alterations than conventional methods could capture, including significant increases in left ONS volume (mean difference ± SEM = 27.22 ± 8.74 mm^3^), automated detection of ON lengthening replicating Wahlin's findings, decreased left globe volume, and lateral expansion of retro‐orbital space boundaries encompassing extraocular muscles and orbital soft tissues (Wahlin et al., 2021). The decreased left globe volume with a forward displacement is likely to reflect posterior globe flattening, as shown in our descriptive analysis (see Figure 5), which produces the characteristic hyperopic refractive shifts observed in ≤48% of long‐duration crew members (Sater et al., 2021; Svoronos et al., 2025). At the same time, lateral retro‐orbital expansion indicates that CSF redistribution affects broader orbital architecture beyond the ONS, potentially reflecting both direct CSF accumulation in orbital tissue compartments and secondary effects from impaired venous drainage owing to loss of the gravitational gradients that usually facilitate venous return through the cavernous sinus and orbital venous plexus (Mampre et al., 2025). Although the observed ocular structural changes are consistent with the effects of increased ICP on the ONS compartment, the asymmetric nature of these changes is notable. Whether manifesting as left‐sided changes (as observed in our study) or right‐sided changes (as predominantly reported in the literature), this asymmetric pattern contradicts the bilateral symmetry expected under uniform ICP elevation (Mader et al., 2017). This asymmetric pattern, instead, might suggest differentiated development of SANS after the initial CSF redistribution effect.

### 4.2 Selective ventricular–ocular morphological relationships: from terrestrial baseline to spaceflight perturbation

To investigate the fundamental mechanisms of cephalad fluid shift potentially driving eye and brain changes via CSF circulation, we conducted a comprehensive correlation analysis between third ventricle volume and ocular structures. We could establish several physiological correlations between third ventricle volume and multiple ocular parameters in healthy control subjects, including globe dimensions, ONS volume, ON length, retro‐orbital widths and left ON tortuosity. Unsurprisingly, these correlations could not be observed at the present raw data resolution of 1 mm isotropic for small cross‐sectional parameters such as ONS head width, ONS 3 mm cross‐sectional area and optical canal area.

These findings provide the first documented baseline ventricle–eye correlation for interpreting spaceflight‐induced ocular changes, establishing that normal CSF dynamics maintain coordinated relationships between central ventricular and orbital compartments. The ONS represents a direct extension of the intracranial CSF space into orbital compartments, and optic nerve sheath diameter has been extensively validated as a biomarker for increased ICP in clinical settings (Jeon et al., 2017; Kimberly et al., 2008). Beyond the ONS, our findings suggest that other orbital structures also serve as indicators of altered CSF dynamics, as evidenced by their baseline correlations with third ventricular volume.

In contrast to baseline correlations across multiple orbital structures, change analysis following spaceflight revealed selective associations: only the magnitude of changes in globe volume and globe length maintained significant negative correlations with changes in the third ventricle. In contrast, previously correlated parameters, i.e., ONS volume, ON length, retro‐orbital width and left ON tortuosity, showed no correlation with the magnitude of third ventricle expansion, despite undergoing significant morphological changes. This structure‐specific correlation pattern provides strong evidence supporting the compartmentalization hypothesis in SANS, because it indicates that spaceflight‐induced CSF redistribution creates differential biomechanical effects across orbital compartments rather than uniform changes in pressure throughout the cranial–orbital system.

Importantly, although the absolute changes in globe length pre‐ to post‐flight (right: −0.13 ± 0.13 mm, *P* = 0.35; left: −0.28 ± 0.20 mm, *P* = 0.18) were not statistically significant, the magnitude of these changes was correlated with third ventricle expansion, demonstrating sustained magnitude‐dependent coupling despite minimal morphological alteration. Our findings showed the same directional shortening as reported by Macias et al. (2020) (0.08 mm axial length reduction in National Aeronautics and Space Administration astronauts). This finding underscores that the magnitude‐dependent relationship, rather than absolute morphological change, serves as the potential indicator of CSF‐mediated biomechanical transmission across intracranial compartments.

The persistence of ventricle–globe correlations following spaceflight suggests that the balance between intraocular pressure and CSF pressure, which determines the translaminar cribrosa pressure difference, remains under sophisticated physiological control (Mirra et al., 2020). In contrast, the loss of correlations between third ventricle volume and ONS volume, ON and retro‐orbital space, together with the observed expansion of the ONS and retro‐orbital width, very probably indicates that these components might develop independent local pressure dynamics during long‐duration spaceflight at the limits of elastic tissue expansion. This divergent pattern is likely to reflect the development of ONS compartmentalization, wherein altered CSF flow dynamics create isolated pressure environments within the ONS compartment. ONS compartment syndrome can develop secondary to increased ICP and impaired CSF flow, leading to localized alterations in pressure that become mechanically decoupled from changes in global CSF pressure (Hao et al., 2020; Killer & Subramanian, 2014). The maintenance of ventricle–globe relationships despite this compartmentalization suggests that globe deformation might remain responsive to central CSF pressure changes, whereas other orbital structures become subject to local pressure dynamics within their respective anatomical compartments.

Given that our study captured chronic adaptations following long‐duration spaceflight rather than the acute temporal dynamics in flight, future research investigating the time course of correlation disruption during microgravity exposure will be essential for understanding the temporal evolution of CSF compartmentalization in SANS.

### 4.3 Acute post‐flight CSF changes: exploratory investigation of differential recovery trajectories

Owing to the limited sample size, we pooled data from all cosmonauts and astronauts to characterize the acute recovery trajectory over the first 14 days post‐flight. Our preliminary analysis revealed distinct compartment‐specific recovery patterns, with relative change of ONS volume showing weak negative correlations (*r* = −0.27 right and *r* = −0.18 left) indicative of a gradual volume reduction to pre‐flight baseline, while relative change of third ventricle volume demonstrated no correlation (*r* = 0.00), suggesting relative stability of expansion during early readaptation.

These differential recovery patterns provide tentative evidence that might inform both competing SANS hypotheses. According to the uniform elevated ICP framework, the findings could reflect differential pressure equilibration kinetics, whereby both compartments experience persistently elevated pressure but exhibit distinct recovery trajectories. The ONS volume reduction towards baseline might occur through different mechanisms from ventricular recovery, potentially related to anatomical differences in CSF drainage pathways and tissue properties, although direct comparative evidence for differential recovery rates remains limited (Jaganathan et al., 2024). In contrast, the maintained ventricular expansion represents slower central pressure relief mechanisms. Alternatively, the compartmentalization hypothesis would interpret these patterns as evidence of independent, non‐uniform pressure dynamics, wherein ONS volume reduction reflects localized pressure normalization through compartment‐specific drainage recovery, possibly the local glymphatic system (Wostyn et al., 2022). At the same time, persistent ventricular expansion indicates distinct pathophysiological mechanisms affecting different CSF spaces independently. The observed weak correlations might suggest that acute recovery involves complex interactions between global pressure changes and compartment‐specific adaptation mechanisms, although the nature of these interactions remains unclear. However, larger sample sizes and extended acute post‐flight monitoring are needed to distinguish definitively between these competing hypotheses for SANS adaptive responses.

### 4.4 Limitations and outlook

Despite providing quantitative evidence for structure‐specific CSF‐mediated relationships, several limitations restrict our ability to characterize fully the underlying mechanisms in SANS and highlight crucial areas for future investigation. We did not and could not gain access to eye examinations and, more importantly, optical coherence tomography data for our cohorts. Our neuroimaging protocol, although optimized for brain morphometry, was not designed explicitly for high‐resolution ocular analysis, resulting in suboptimal spatial resolution and retro‐orbital tissue contrast with potential partial volume effects. We feel that the findings for the available 1 mm data are still remarkable. All of this was achieved mostly through the Advanced Normalisation Tools (ANTs) template‐building methods. However, future analysis with the same available methods (MReye‐Seg; https://github.com/Eulenburg‐LMU/MReye‐Seg) would certainly benefit from dedicated orbital imaging protocols with higher resolution (e.g., <0.75 mm isotropic) and multi‐contrast T_1_/T_2_/T_2_*‐weighted sequences optimized for fat suppression, CSF visualization and orbital soft tissue differentiation.

More of a logistical obstacle than a real limitation is the lack of densely sampled temporal post‐flight data from the first 24 h after returning to Earth. This period most probably represents the most dynamic phase of circulatory readaptation to Earth's gravity. This study design, owing to logistical constraints, hampers almost all long‐duration spaceflight investigations to date and introduces a variability in post‐flight data collection time points. For the cohort of cosmonauts in our study, the earliest post‐flight scan was taken on day 5 (the latest on day 13), whereas for the cohort of astronauts, it was possible to measure some on day 1. The aim here should be to increase the neuroimaging measurement frequency of spacefarers at the earliest beginning of this readaptation phase. Such protocols could very well be standardized in space health surveillance and research across space agencies.

## 5 CONCLUSION

This study demonstrates structure‐specific relationships consistent with CSF‐mediated brain–eye coupling as a mechanism in SANS, providing quantitative evidence that microgravity creates differential biomechanical effects across orbital compartments through selective coupling of the third ventricle and the globe, while dissociating from ON, ONS and other retro‐orbital structures. Critically, change analysis revealed selective magnitude‐dependent associations: third ventricle expansion was correlated exclusively with globe changes, whereas ONS, ON and retro‐orbital parameters showed no correlation with ventricular dynamics despite undergoing significant morphological alterations. Together with the post‐flight left‐side dominance changes observed in the ON length, ONS and globe, these findings directly support CSF compartmentalization owing to the interruption of CSF dynamics as a potential SANS mechanism, reframing the syndrome from an isolated ocular pathology to a systemic brain–eye physiological disruption. We hope it provides critical mechanistic insights for developing targeted countermeasures essential for maintaining visual health of the crew during future exploration missions to the Moon, Mars and beyond. We now consider the majority of observed structural changes in the brain and eye to be inherently linked and to reflect the cranial microgravity response to cephalad fluid shift (Seidler et al., 2024; Zu Eulenburg et al., 2025).

## AUTHOR CONTRIBUTIONS

Ge Tang conceptualized the study, performed the data analysis and drafted the manuscript. Peter zu Eulenburg supervised the study, contributed to the initial conception and conceptualization of the study, wrote part of the Materials and Methods section and provided critical revisions to the manuscript. Seyed‐Ahmad Ahmadi contributed to the data analysis. Steven Jillings, Ben Jeurissen, Elena Tomilovskaya, Inna Nosikova, Alexandra Ryabova, Ekaterina Pechenkova, Viktor Petrovichev, Ilya Rukavishnikov, Stefan Sunaert, Paul M. Parizel, Lyudmila Makovskaya, Valentin Sinitsyn, Jan Sijbers, Jitka Annen, Steven Laureys, Angelique Van Ombergen and Floris L. Wuyts participated in data collection and acquisition. All authors revised the manuscript critically for important intellectual content. All authors have read and approved the final version of the manuscript and agree to be accountable for all aspects of the work in ensuring that questions related to the accuracy or integrity of any part of the work are appropriately investigated and resolved. All persons designated as authors qualify for authorship, and all those who qualify for authorship are listed.

## CONFLICT OF INTEREST

Peter zu Eulenburg is a senior editor of *Experimental Physiology*. The other authors declare that they have no competing interests.

## Supporting information



Supplementary Figure 1. Investigation of the effect of postflight scanning time point on ONS and third ventricle volume.
Supplementary Figure 2. The visualisation of segmentations and landmarks on the raw image of one representative subject.
Supplementary Figure 3. Heatmap depicting correlations between ocular and brain morphometrics in the terrestrial controls after controlling for total intracranial volume (TIV)
Supplementary Table 1. The intraclass correlation coefficient (ICC) of the eye morphometrics and the intracranial ventricle volume measurements in the pooled data from cosmonauts and controls (obtained from two consecutive MRI scans around 30 minutes apart).
Supplementary Table 2. Ocular and retroorbital structure pre‐post flight differences for the ESA Astronaut cohort.
Supplementary Table 3. Pre‐ and post‐flight changes in ocular metrics among long‐duration spaceflight cosmonauts and controls
Supplementary Table 4. Correlation between third ventricle volume changes and ocular metric changes in long‐duration spaceflight cosmonauts

## Data Availability

Please contact Floris L. Wuyts (floris.wuyts@uantwerpen.be) and Elena Tomilovskaya (finegold@yandex.ru) for questions concerning ROS cosmonaut and ESA astronaut data. The pipeline code for ocular segmentation is freely available at https://github.com/Eulenburg‐LMU/MReye‐Seg
