# Supplementary material for: Physiological cerebrospinal fluid interactions between brain and eye structures are altered after long‐duration spaceflight
Source: Exp Physiol. 2026 Jan 16:10.1113/EP093112. Online ahead of print. doi: 10.1113/EP093112 (PMC13394203; doi:10.1113/EP093112)
Supplement: Supplementary file 1 — Supplementary Figure 1. Investigation of the effect of postflight scanning time point on ONS and third ventricle volume. Supplementary Figure 2. The visualisation of segmentations and landmarks on the raw image of one representative subject. Supplementary Figure 3. Heatmap depicting correlations between ocular and brain morphometrics in the terrestrial controls after controlling for total intracranial volume (TIV) Supplementary Table 1. The intraclass correlation coefficient (ICC) of the eye morphometrics and the intracranial ventricle volume measurements in the pooled data from cosmonauts and controls (obtained from two consecutive MRI scans around 30 minutes apart). Supplementary Table 2. Ocular and retroorbital structure pre‐post flight differences for the ESA Astronaut cohort. Supplementary Table 3. Pre‐ and post‐flight changes in ocular metrics among long‐duration spaceflight cosmonauts and controls Supplementary Table 4. Correlation between third ventricle volume changes and ocular metric changes in long‐duration spaceflight cosmonauts [file EPH-9999-0-s001.docx]

**
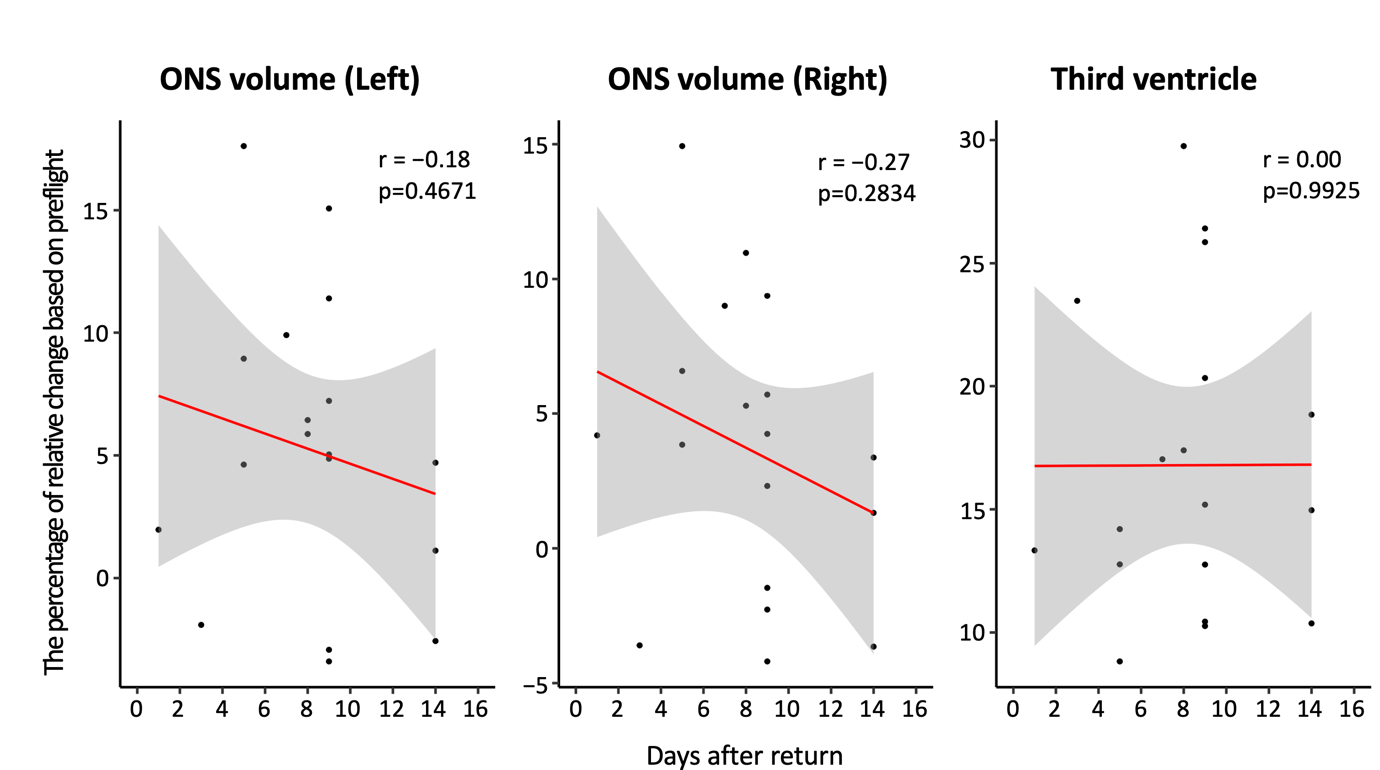
 Supplementary Figure 1. Investigation of the effect of postflight scanning time point on ONS and third ventricle volume.** The above plot shows the dynamic volume changes within the ONS (Left/Right), and the third ventricle, concerning the time point of MRI data acquisition in the two weeks after return to the earth. The y-axis shows the relative volume change of the compartments regarding the preflight volume. In this preliminary analysis, ONS volume increase appears to be affected by the time point of scanning within the first two weeks after return, while the third ventricle volume remains substantially (+17% in our study) enlarged for the same period after the return from a long-duration mission. However, the depicted time curves are not significant and thus warrant more research before we can draw any conclusions from them. But the depicted timelines illustrate in our opinion the need for densely sampled serial neuroimaging of the eye and brain in order to better model the brain- and eye-structural recovery phase after LDSF.


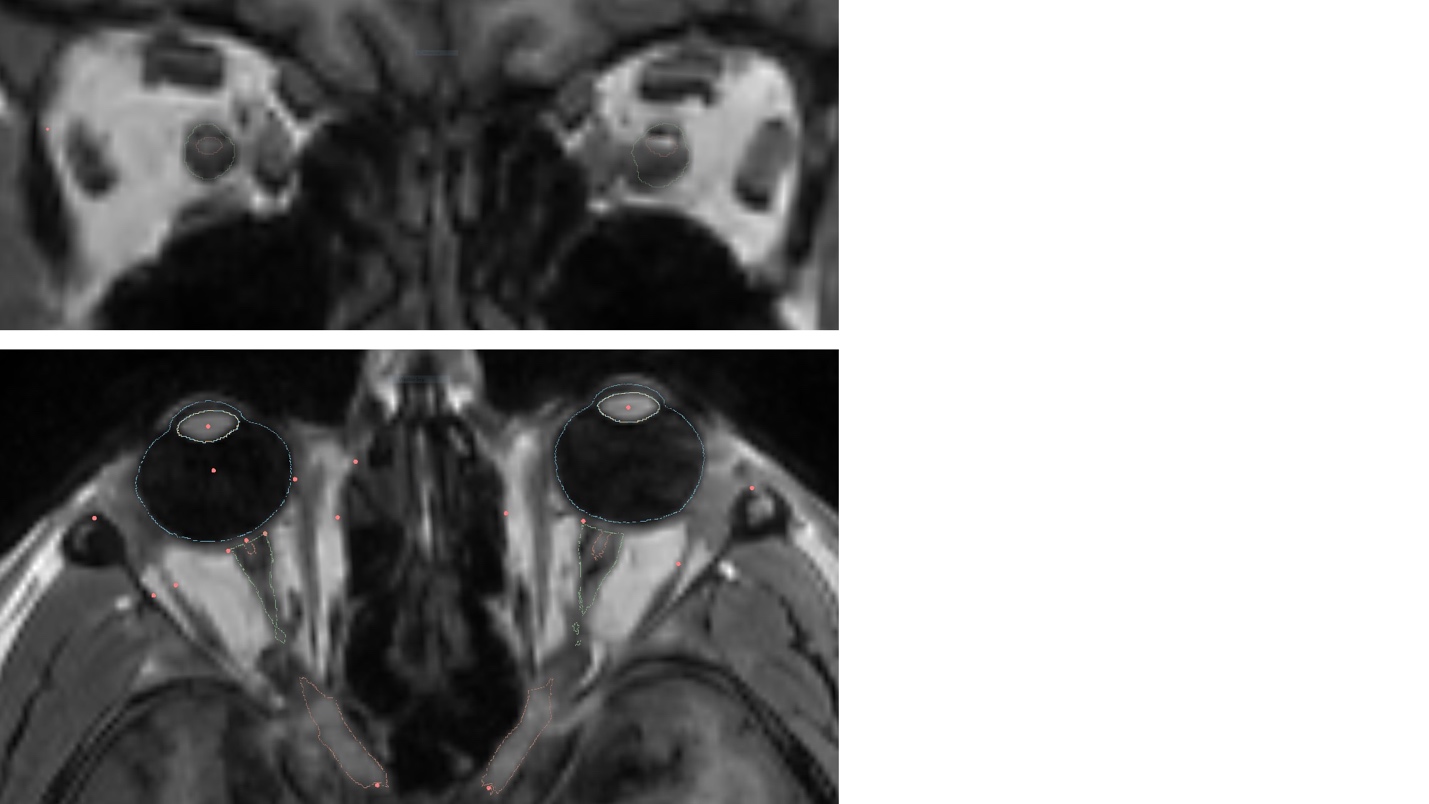


**Supplementary Figure 2. The visualisation of segmentations and landmarks on the raw image of one representative subject.**


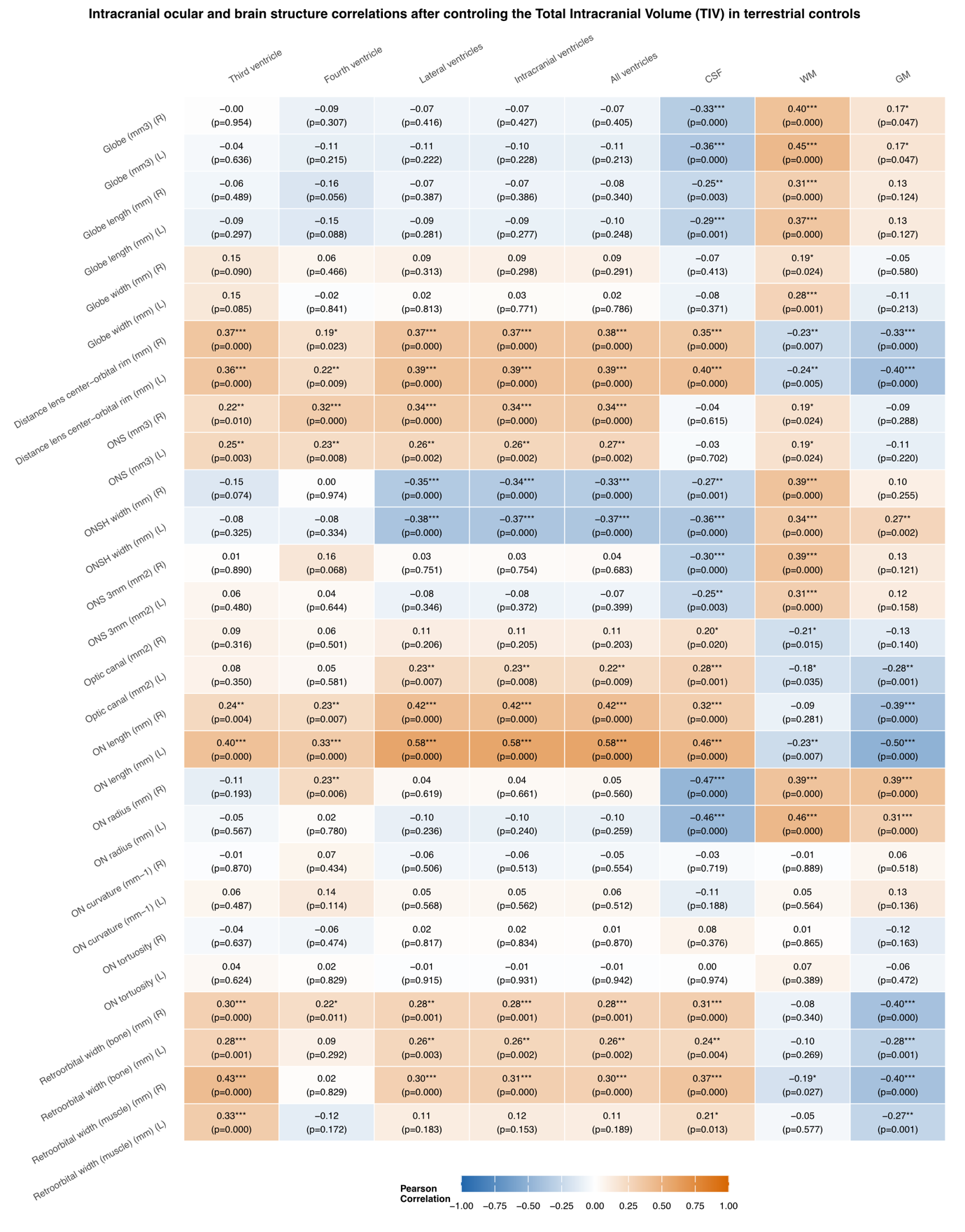


**Supplementary Figure 3. Heatmap depicting correlations between ocular and brain morphometrics in the terrestrial controls after controlling for total intracranial volume (TIV)**

**Supplementary Table 1. The intraclass correlation coefficient (ICC) of the eye morphometrics and the intracranial ventricle volume measurements in the pooled data from cosmonauts and controls (obtained from two consecutive MRI scans around 30 minutes apart).**

|  | **ICC** | **ICC p value** | **Mean difference*** |
| --- | --- | --- | --- |
| **Third ventricle (cm3)** | **0.99** | **<0.0001** | **-0.01** |
| **Fourth ventricle (cm3)** | **1** | **<0.0001** | **0** |
| **Lateral ventricle (cm3)** | **1** | **<0.0001** | **0.01** |
| **Intracranial ventricles (cm3)** | **1** | **<0.0001** | **0** |
| **All ventricles (cm3)** | **1** | **<0.0001** | **0** |
| **CSF (cm3)** | **0.95** | **<0.0001** | **3.66** |
| **WM (cm3)** | **1** | **<0.0001** | **-0.58** |
| **GM (cm3)** | **0.99** | **<0.0001** | **-1.92** |
| **White Matter Hyperintensities** | **0.97** | **<0.0001** | **0** |
| **Whole Brain volume (cm3)** | **0.98** | **<0.0001** | **1.15** |
| **Globe (cm3) (R)** | **0.98** | **<0.0001** | **0.02** |
| **Globe (cm3) (L)** | **0.97** | **<0.0001** | **0.04** |
| **Globe length (mm) (R)** | **0.85** | **<0.0001** | **0.06** |
| **Globe length (mm) (L)** | **0.79** | **<0.0001** | **0.04** |
| **Globe width (mm) (R)** | **0.97** | **<0.0001** | **-0.02** |
| **Globe width (mm) (L)** | **0.94** | **<0.0001** | **0.03** |
| **Distance lens center-orbital rim (mm) (R)** | **0.96** | **<0.0001** | **-0.21** |
| **Distance lens center-orbital rim (mm) (L)** | **0.95** | **<0.0001** | **-0.18** |
| **ONS (cm3) (R)** | **0.81** | **0.0004** | **-0.03** |
| **ONS (cm3) (L)** | **0.71** | **0.0005** | **-0.04** |
| **ONSH width (mm) (R)** | **0.57** | **<0.0001** | **-0.11** |
| **ONSH width (mm) (L)** | **0.71** | **<0.0001** | **-0.18** |
| **ONS 3mm (mm2) (R)** | **0.57** | **0.0003** | **-2.51** |
| **ONS 3mm (mm2) (L)** | **0.58** | **<0.0001** | **-2.57** |
| **Optic canal (mm2) (R)** | **0.88** | **<0.0001** | **0.06** |
| **Optic canal (mm2) (L)** | **0.87** | **<0.0001** | **-0.03** |
| **ON length (mm) (R)** | **0.96** | **<0.0001** | **-0.2** |
| **ON length (mm) (L)** | **0.96** | **<0.0001** | **-0.16** |
| **ON radius (mm) (R)** | **0.74** | **<0.0001** | **-0.02** |
| **ON radius (mm) (L)** | **0.73** | **<0.0001** | **-0.02** |
| **ON curvature (mm‑1) (R)** | **0.19** | **0.0539** | **-0.01** |
| **ON curvature (mm‑1) (L)** | **0.29** | **0.0096** | **0.01** |
| **ON tortuosity (R)** | **0.90** | **<0.0001** | **0** |
| **ON tortuosity (L)** | **0.89** | **<0.0001** | **0** |
| **retroorbital width (bone) (mm) (R)** | **0.97** | **<0.0001** | **-0.02** |
| **Retroorbital width (bone) (mm) (L)** | **0.98** | **<0.0001** | **-0.02** |
| **retroorbital width (muscle) (mm) (R)** | **0.89** | **<0.0001** | **0.02** |
| **retroorbital width (muscle) (mm) (L)** | **0.90** | **<0.0001** | **-0.01** |

* The mean difference is the mean of the differences between two consecutive within-subject MRI scans

**Supplementary Table 2. Ocular and retroorbital structure pre-post flight differences for the ESA Astronaut cohort.**

|  | **Mean difference** | **SE** |
| --- | --- | --- |
| **Globe (mm3) (R)** | **36.76** | **57.78** |
| **Globe (mm3) (L)** | **44.79** | **95.09** |
| **Globe length (mm) (R)** | **-0.31** | **0.28** |
| **Globe length (mm) (L)** | **-0.27** | **0.29** |
| **Globe width (mm) (R)** | **0.01** | **0.18** |
| **Globe width (mm) (L)** | **0.10** | **0.14** |
| **ONS (mm3) (R)** | **35.16** | **17.06** |
| **ONS (mm3) (L)** | **37.65** | **19.56** |
| **ONSH width (mm) (R)** | **-0.03** | **0.2** |
| **ONSH width (mm) (L)** | **0.21** | **0.07** |
| **Optic canal (mm2) (R)** | **0.51** | **0.17** |
| **Optic canal (mm2) (L)** | **0.71** | **0.3** |
| **retroorbital space width (bone) (mm) (R)** | **0.12** | **0.13** |
| **retroorbital space width (bone) (mm) (L)** | **0.10** | **0.07** |
| **retroorbital space width (muscle) (mm) (R)** | **-0.15** | **0.09** |
| **retroorbital space width (muscle) (mm) (L)** | **-0.02** | **0.12** |

**Supplementary Table 3. Pre- and post-flight changes in ocular metrics among long-duration spaceflight cosmonauts and controls**

|  | **Spacefarer mean difference** | **Spacefarer lower ci** | **Spacefarer upper ci** | **Spacefarer se** | **Spacefarer cohen’s d** | **Spacefarer t** | **Spacefarer p value** | **Spacefarer sig** | **Control mean difference** | **Control lower ci** | **Control upper ci** | **Control se** | **Control cohen’s d** | **Control t** | **Control p value** | **Control sig** |
| --- | --- | --- | --- | --- | --- | --- | --- | --- | --- | --- | --- | --- | --- | --- | --- | --- |
| GM | -8.387 | -12.016 | -4.758 | 1.666 | -1.4 | -5.035 | 0.0003 | *** | 2.81 | -3.162 | 8.781 | 2.784 | 0.26 | 1.009 | 0.33 |  |
| WM | 1.579 | -2.211 | 5.369 | 1.739 | 0.25 | 0.908 | 0.3819 |  | 2.536 | 0.757 | 4.315 | 0.829 | 0.79 | 3.058 | 0.0085 | ** |
| CSF | 9.872 | 1.311 | 18.433 | 3.929 | 0.7 | 2.512 | 0.0273 | * | -1.827 | -9.997 | 6.342 | 3.809 | -0.12 | -0.48 | 0.6388 |  |
| All ventricles | 1.404 | 0.819 | 1.989 | 0.269 | 1.45 | 5.228 | 0.0002 | *** | -0.167 | -0.525 | 0.192 | 0.167 | -0.26 | -0.997 | 0.3359 |  |
| Intracranial ventricles | 1.404 | 0.839 | 1.97 | 0.259 | 1.5 | 5.414 | 0.0002 | *** | -0.142 | -0.491 | 0.207 | 0.163 | -0.22 | -0.871 | 0.3984 |  |
| Lateral ventricle | 1.294 | 0.745 | 1.843 | 0.252 | 1.42 | 5.132 | 0.0002 | *** | -0.134 | -0.473 | 0.205 | 0.158 | -0.22 | -0.847 | 0.4115 |  |
| Fourth ventricle | -0.001 | -0.026 | 0.025 | 0.012 | -0.02 | -0.061 | 0.952 |  | -0.025 | -0.049 | 0 | 0.011 | -0.56 | -2.176 | 0.0472 | * |
| Third ventricle | 0.111 | 0.089 | 0.132 | 0.01 | 3.1 | 11.193 | 0 | *** | -0.008 | -0.024 | 0.009 | 0.008 | -0.27 | -1.027 | 0.322 |  |
| Globe (R) | -71.29 | -156.213 | 13.633 | 38.977 | -0.51 | -1.829 | 0.0923 |  | -38.001 | -92.571 | 16.569 | 25.443 | -0.39 | -1.494 | 0.1575 |  |
| Globe (L) | -112.155 | -224.358 | 0.048 | 51.497 | -0.6 | -2.178 | 0.0501 |  | -12.664 | -71.597 | 46.269 | 27.477 | -0.12 | -0.461 | 0.652 |  |
| Globe length (R) | -0.127 | -0.412 | 0.159 | 0.131 | -0.27 | -0.967 | 0.3525 |  | -0.001 | -0.132 | 0.129 | 0.061 | -0.01 | -0.023 | 0.9823 |  |
| Globe length (L) | -0.279 | -0.71 | 0.153 | 0.198 | -0.39 | -1.408 | 0.1846 |  | 0.032 | -0.064 | 0.128 | 0.045 | 0.18 | 0.71 | 0.4895 |  |
| Globe width (R) | -0.021 | -0.177 | 0.134 | 0.071 | -0.08 | -0.3 | 0.7695 |  | -0.012 | -0.111 | 0.088 | 0.046 | -0.07 | -0.256 | 0.8019 |  |
| Globe width (L) | -0.025 | -0.196 | 0.146 | 0.078 | -0.09 | -0.32 | 0.7543 |  | 0.021 | -0.086 | 0.129 | 0.05 | 0.11 | 0.425 | 0.677 |  |
| Distance lens center-orbital rim (R) | 0.22 | -0.048 | 0.488 | 0.122 | 0.52 | 1.81 | 0.0976 |  | -0.023 | -0.235 | 0.189 | 0.099 | -0.06 | -0.237 | 0.8161 |  |
| Distance lens center-orbital rim (L) | 0.434 | 0.101 | 0.768 | 0.151 | 0.83 | 2.87 | 0.0152 | * | 0.112 | -0.121 | 0.345 | 0.109 | 0.27 | 1.032 | 0.3196 |  |
| ONS (R) | 11.928 | -1.305 | 25.161 | 6.074 | 0.54 | 1.964 | 0.0731 |  | 6.223 | -11.767 | 24.213 | 8.388 | 0.19 | 0.742 | 0.4704 |  |
| ONS (L) | 27.222 | 8.174 | 46.27 | 8.742 | 0.86 | 3.114 | 0.009 | ** | 8.097 | -11.441 | 27.635 | 9.109 | 0.23 | 0.889 | 0.3891 |  |
| ONSH width (R) | 0.079 | -0.046 | 0.204 | 0.057 | 0.38 | 1.377 | 0.1936 |  | 0.032 | -0.165 | 0.23 | 0.092 | 0.09 | 0.351 | 0.731 |  |
| ONSH width (L) | 0.103 | -0.111 | 0.317 | 0.098 | 0.29 | 1.05 | 0.3144 |  | 0.017 | -0.181 | 0.216 | 0.092 | 0.05 | 0.187 | 0.8547 |  |
| ONS 3mm (R) | 0.429 | -1.52 | 2.378 | 0.895 | 0.13 | 0.48 | 0.6401 |  | 0.839 | -1.002 | 2.679 | 0.858 | 0.25 | 0.977 | 0.345 |  |
| ONS 3mm (L) | 0.012 | -4.437 | 4.462 | 2.042 | 0 | 0.006 | 0.9953 |  | 0.909 | -1.151 | 2.969 | 0.96 | 0.24 | 0.946 | 0.3599 |  |
| Optic canal (R) | -0.049 | -0.435 | 0.338 | 0.177 | -0.08 | -0.275 | 0.7881 |  | 0.443 | -0.014 | 0.9 | 0.213 | 0.54 | 2.079 | 0.0565 |  |
| Optic canal (L) | 0.056 | -0.123 | 0.234 | 0.082 | 0.19 | 0.679 | 0.5102 |  | 0.11 | -0.121 | 0.34 | 0.107 | 0.26 | 1.02 | 0.3251 |  |
| ON length (R) | 0.378 | 0.055 | 0.7 | 0.148 | 0.71 | 2.553 | 0.0253 | * | -0.044 | -0.352 | 0.264 | 0.144 | -0.08 | -0.305 | 0.7649 |  |
| ON length (L) | 0.466 | 0.087 | 0.845 | 0.174 | 0.74 | 2.682 | 0.02 | * | 0.133 | -0.109 | 0.375 | 0.113 | 0.3 | 1.178 | 0.2582 |  |
| ON radius (R) | 0.005 | -0.015 | 0.024 | 0.009 | 0.15 | 0.555 | 0.5891 |  | 0.001 | -0.013 | 0.015 | 0.007 | 0.04 | 0.138 | 0.8925 |  |
| ON radius (L) | 0.003 | -0.021 | 0.027 | 0.011 | 0.08 | 0.282 | 0.783 |  | 0.005 | -0.01 | 0.019 | 0.007 | 0.17 | 0.664 | 0.5175 |  |
| ON curvature (R) | 0.007 | -0.01 | 0.024 | 0.008 | 0.25 | 0.908 | 0.3817 |  | 0.005 | -0.012 | 0.021 | 0.008 | 0.16 | 0.625 | 0.5419 |  |
| ON curvature (L) | 0 | -0.029 | 0.029 | 0.013 | 0 | 0.016 | 0.9871 |  | -0.004 | -0.02 | 0.012 | 0.008 | -0.14 | -0.549 | 0.5915 |  |
| ON tortuosity (R) | -0.001 | -0.005 | 0.004 | 0.002 | -0.08 | -0.28 | 0.7843 |  | 0.003 | -0.002 | 0.007 | 0.002 | 0.33 | 1.294 | 0.2167 |  |
| ON tortuosity (L) | 0 | -0.005 | 0.006 | 0.003 | 0.04 | 0.136 | 0.8943 |  | 0 | -0.003 | 0.004 | 0.002 | 0.04 | 0.148 | 0.8847 |  |
| Retroorbital width (bone) (R) | 0.204 | 0.082 | 0.326 | 0.056 | 1.01 | 3.65 | 0.0033 | ** | -0.021 | -0.148 | 0.106 | 0.059 | -0.09 | -0.362 | 0.7228 |  |
| Retroorbital width (bone) (L) | 0.156 | 0.054 | 0.258 | 0.047 | 0.93 | 3.345 | 0.0058 | ** | -0.091 | -0.179 | -0.003 | 0.041 | -0.57 | -2.22 | 0.0434 | * |
| Retroorbital width (muscle) (R) | 0.14 | 0.018 | 0.261 | 0.056 | 0.69 | 2.503 | 0.0277 | * | 0.031 | -0.092 | 0.155 | 0.057 | 0.14 | 0.545 | 0.5941 |  |
| Retroorbital width (muscle) (L) | 0.148 | -0.002 | 0.298 | 0.069 | 0.6 | 2.15 | 0.0526 |  | 0.127 | -0.013 | 0.266 | 0.065 | 0.5 | 1.948 | 0.0717 |  |

**Supplementary Table 4. Correlation between third ventricle volume changes and ocular metric changes in long-duration spaceflight cosmonauts**

|  | **Correlation** | **Lower ci** | **Upper ci** | **P value** | **Significance** |
| --- | --- | --- | --- | --- | --- |
| Globe (R) | -0.583 | -0.858 | -0.047 | 0.0366 | * |
| Globe (L) | -0.547 | -0.844 | 0.005 | 0.0528 |  |
| Globe length (R) | -0.599 | -0.865 | -0.072 | 0.0304 | * |
| Globe length (L) | -0.577 | -0.856 | -0.038 | 0.0391 | * |
| Globe width (R) | -0.359 | -0.76 | 0.239 | 0.2279 |  |
| Globe width (L) | -0.086 | -0.608 | 0.488 | 0.78 |  |
| Distance lens center-orbital rim (R) | -0.467 | -0.809 | 0.114 | 0.108 |  |
| Distance lens center-orbital rim (L) | -0.265 | -0.712 | 0.335 | 0.3823 |  |
| ONS (R) | -0.295 | -0.728 | 0.305 | 0.3274 |  |
| ONS (L) | 0.091 | -0.484 | 0.611 | 0.7682 |  |
| ONSH width (R) | 0.148 | -0.439 | 0.646 | 0.6289 |  |
| ONSH width (L) | 0.516 | -0.049 | 0.831 | 0.071 |  |
| ONS 3mm (R) | -0.161 | -0.654 | 0.428 | 0.6004 |  |
| ONS 3mm (L) | -0.191 | -0.671 | 0.403 | 0.5324 |  |
| Optic canal (R) | 0.167 | -0.423 | 0.658 | 0.5854 |  |
| Optic canal (L) | -0.056 | -0.589 | 0.511 | 0.8553 |  |
| ON length (R) | -0.099 | -0.616 | 0.478 | 0.7484 |  |
| ON length (L) | 0.474 | -0.104 | 0.813 | 0.1019 |  |
| ON radius (R) | -0.212 | -0.683 | 0.384 | 0.4869 |  |
| ON radius (L) | -0.054 | -0.588 | 0.512 | 0.8607 |  |
| ON curvature (R) | -0.519 | -0.832 | 0.045 | 0.0694 |  |
| ON curvature (L) | 0.126 | -0.456 | 0.633 | 0.6807 |  |
| ON tortuosity (R) | -0.08 | -0.604 | 0.493 | 0.7958 |  |
| ON tortuosity (L) | 0.469 | -0.111 | 0.811 | 0.106 |  |
| Retroorbital width (bone) (R) | 0.253 | -0.347 | 0.705 | 0.4049 |  |
| Retroorbital width (bone) (L) | 0.285 | -0.316 | 0.722 | 0.3454 |  |
| Retroorbital width (muscle) (R) | -0.051 | -0.585 | 0.515 | 0.8692 |  |
| Retroorbital width (muscle) (L) | 0.54 | -0.016 | 0.841 | 0.0568 |  |
